# Mesenchymal stromal cells in myeloid malignancies: Immunotherapeutic opportunities

**DOI:** 10.1016/j.heliyon.2024.e25081

**Published:** 2024-01-22

**Authors:** Milica Vukotić, Suncica Kapor, Felipe Simon, Vladan Cokic, Juan F. Santibanez

**Affiliations:** a Molecular Oncology Group, Institute for Medical Research, University of Belgrade, Belgrade, Serbia; b Department of Hematology, Clinical Hospital Center “Dr. Dragisa Misovic-Dedinje,” University of Belgrade, Serbia; c Laboratory of Integrative Physiopathology, Faculty of Life Sciences, Universidad Andres Bello, Santiago, Chile; d Millennium Institute on Immunology and Immunotherapy, Santiago, Chile; e Millennium Nucleus of Ion Channel-Associated Diseases, Universidad de Chile, Santiago, Chile; f Centro Integrativo de Biología y Química Aplicada (CIBQA), Universidad Bernardo O'Higgins, Santiago, Chile

**Keywords:** Myeloid malignancies, Myeloid cells, Mesenchymal stromal cells, Cell differentiation, T-cell immunosuppression therapy

## Abstract

Myeloid malignancies are clonal disorders of the progenitor cells or hematopoietic stem cells, including acute myeloid leukemia, myelodysplastic syndromes, myeloproliferative malignancies, and chronic myelomonocytic leukemia. Myeloid neoplastic cells affect the proliferation and differentiation of other hematopoietic lineages in the bone marrow and peripheral blood, leading to severe and life-threatening complications. Mesenchymal stromal cells (MSCs) residing in the bone marrow exert immunosuppressive functions by suppressing innate and adaptive immune systems, thus creating a supportive and tolerant microenvironment for myeloid malignancy progression. This review summarizes the significant features of MSCs in myeloid malignancies, including their role in regulating cell growth, cell death, and antineoplastic resistance, in addition to their immunosuppressive contributions. Understanding the implications of MSCs in myeloid malignancies could pave the path for potential use in immunotherapy.

## 1 Introduction

Hematopoietic stem cells (HSCs) are undifferentiated and multipotent precursor cells crucial in continually regenerating the blood cell pool throughout an organism's life [[1], [2], [3]]. A combination of cell-intrinsic characteristics of the HSCs and extrinsic signals from the bone marrow microenvironment (BMME) intricately controls the production of blood. When this delicate balance is disrupted, it can lead to the development of hematopoietic malignancies. The bone marrow (BM) niche is a complex environment composed of various cellular populations, including mesenchymal stromal cells (MSCs) and their descendants, immunological cell compartments, vascular networks, nerves, and cytokines, among others [[4], [5], [6]].

Myeloid malignancies, also known as myeloid neoplasms, are a group of diverse hematological cancers that develop due to the unrestricted proliferation of neoplastic cells. The pathogenesis of myeloid malignancies involves genetic and epigenetic changes within the neoplastic population and a dysfunctional bone marrow stroma that can facilitate the neoplastic process. The interaction between molecular and cellular components in the BMME contributes to neoplastic transformation by affecting critical mechanisms involved in cell growth, viability, and immune surveillance [7,8].

MSCs have been identified as crucial BMME players with a hematopoiesis role [9,10]. Depending on the context, MSCs can exhibit prooncogenic or antitumor properties. MSCs contribute to myeloid malignancies' progression by generating microenvironment (TME) that may promote cell malignancy, inhibiting immune cell proliferation or activation, including B cells, T cells, dendritic cells, Natural killer (NK) cells, and macrophages [11]. Another important pro-tumorigenic activity of MSC is promoting resistance to chemotherapy. Oppositely to the well-described role of MSC in tumor progression, evidence supports their anti-tumorigenic properties, mainly via induction of cell cycle arrest and apoptosis. This review focuses on the current understanding of how human MSCs are actively involved in myeloid disease development, such as acute myeloid leukemia (AML), myelodysplastic syndromes (MDS), and myeloproliferative neoplasms (MPN). This review also presents new strategies and perspectives to develop or improve cancer management therapies and ultimately increase patients' life expectancy.

## 2 Myeloid malignancies

Myeloid malignancies result from genetic mutations conducive to the clonal expansion of abnormal cells in the myeloid lineage, including HSCs and immature and mature blood cells. These mutations alter cell proliferation, maturation, and differentiation, accumulating abnormal cells and developing malignancies [12,13]. The 2017 update of the World Health Organization (WHO) classification of myeloid malignancies categorizes them into different types, such as AML, MDS, BCR-ABL1/Philadelphia (Ph) positive and negative MPN, and MDS/MPN with hybrid characteristics [[14], [15], [16]].

### 2.1 Acute myeloid leukemia (AML)

AML is a malignant disorder characterized by the proliferation of immature myeloid blasts in the bone marrow and peripheral blood [17,18]. AML occurs due to hematopoietic progenitor transformation, accumulating abnormal myeloid cells that cannot proliferate or differentiate properly [19,20].

**Fig. 1:**
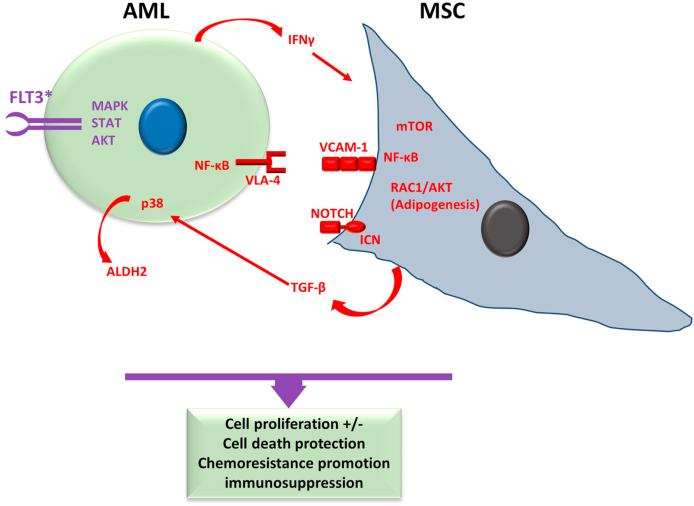
**Main features of Acute Myeloid Leukemia and Mesenchymal Stroma Cells:** The most common mutation in AML is a mutation in the Flt3 gene, which is also a marker of poor prognosis, and provokes intracellular MAPK, STAT, and AKT signals. Meanwhile, MSCs can induce or reduce malignant cell proliferation and promote chemoresistance along with promoting immunosuppression protecting neoplastic cells from immunosurveillance. For more details, see the text. Purple indicates the main molecular characteristic of AML, and red indicates the main aspects of MSCs in interaction with the neoplastic cells. MSC, mesenchymal stroma cells; AML, acute myeloid leukemia; FLT3, FMS-like tyrosine kinase 3; TGF-β, Transforming growth factor-beta; ALDH2, aldehyde dehydrogenase-2; IFNγ, interferon-gamma; VCAM-1, vascular cell adhesion molecule-1; VLA-4, very late antigen-4; ICN, intracellular domain of Notch; MAPK, mitogen-activated protein kinases. (For interpretation of the references to colour in this figure legend, the reader is referred to the Web version of this article.)

AML is propitiated by mutational oncogenic alteration in myeloid progenitor cells, affecting the function of growth factors, signaling pathway receptors, and downstream effectors, causing acute pancytopenia, severe bleeding, and infection [18,20,21]. Molecularly, AML is mainly characterized by Nuclear-cytoplasmic shuttling phosphoprotein (NPM1) mutation and by FMS-like tyrosine kinase 3 (Flt3), a Class III receptor tyrosine kinase, genetic alterations that include internal tandem duplication (FLT3-ITD) tyrosine kinase domain amino acid substitution (FLT3-TKD) and promising molecular therapeutics target [22]. A gain-of-function due to alteration in the Flt3 gene activates other intracellular signal pathways, including mitogen-activated protein kinases (MAPK), signal transducer and activator of transcription (STAT), and AKT crucial in inducing an uncontrolled cell proliferation and cell death resistance in AML (Fig. 1) [23]. Additionally, immunosurveillance escape is a significant feature of AML [24,25]. Systematic analyses of the mechanisms of AML progression are essential for developing novel therapeutic strategies for AML.

### 2.2 Myelodysplastic syndrome (MDS)

**Fig. 2:**
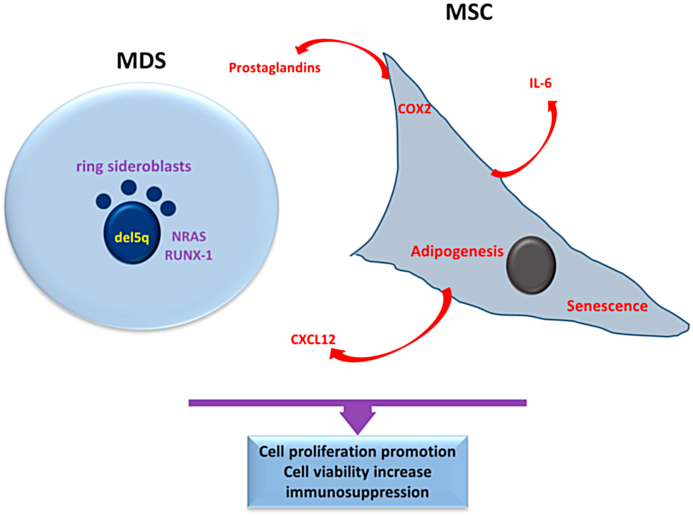
**Main features of Myelodysplastic Syndrome and Mesenchymal Stroma Cells:** the most common aspect of MDS classification is the presence of ring sideroblasts and chromosomal abnormalities, such as del5q, accompanied by NRAS and RUNX1 mutations, among others. In turn, MSC promotes MDS cell proliferation and viability and produces CXCL12 chemokine related to the survival/anti-apoptotic effects on MDS cells. Also, MSC exhibits increased adipogenesis differentiation as well as increased senescence frequency. Moreover, MSCs secreted prostaglandins that collaborated with their immunosuppression activities. Purple indicates the main molecular characteristic of AML, and red indicates the main aspects of MSCs in interaction with the neoplastic cells. MDS, myelodysplastic syndrome, MSC, mesenchymal stroma cells; COX2, cyclooxygenase-2; CXCL12, C-X-C motif chemokine ligand 12, RUNX-1, runt-related transcription factor 1. (For interpretation of the references to colour in this figure legend, the reader is referred to the Web version of this article.)

MDS is a neoplasm type in which HSCs are affected, and myelopoiesis becomes dysplastic, leading to pancytopenia. The pathological characterization of MDS is determined by the amount of dysplastic myeloid cell lineages (single- or multilineage), the presence of ring sideroblasts (RS-MDS), a long arm of chromosome 5 (del(5q) interstitial deletion, or an excess of blasts in bone marrow or peripheral blood (MDS with excess blasts) (Fig. 2) [[26], [27], [28], [29], [30]]. Cytopenia occurs due to increased apoptosis or phagocytosis and decreased cell differentiation, leading to an increased risk of blood loss or microorganism diseases. These complications can be the primary basis of mortality in MDS patients [[31], [32], [33], [34]]. This myeloid malignancy is the most frequently diagnosed disorder, affecting primarily older adults. Around 30%–45 % of patients progress to AML [[34], [35], [36]].

Genetic irregularities such as del5q, del7q, t(7; 17), NRAS, and runt-related transcription factor (RUNX)-1 gene mutations may be identified during the medical analysis or the clinical course. Recent research has shown that these genetic abnormalities play a critical role in diagnosing, categorizing, and predicting drug response and prognosis of MDS [37,38]. A systemic and local inflammatory landscape and immune aberrant functions are critical aspects of the pathogenesis of MDS. MDS progenitor cells are susceptible to apoptosis because of the increased expression of death receptors (such as Fas and TRAIL-R) and aberrant caspase activation. MDS is associated with the over-expression of suppressive cytokines like tumor necrosis factor-alpha (TNF-α), interferon-gamma (IFN-γ), and transforming growth factor (TGF)-β, and the over-expression of toll-like receptors (TLRs). Also, It has been observed that cytolytic T-cells are hyper-activated [[39], [40], [41]].

### 2.3 Myeloproliferative neoplasms (MPN)

MPNs are mainly identified by an abnormally increased number of myeloid cells entering the bloodstream [42]. The main types of MPNs are the BCR-ABL1/Philadelphia (Ph) positive chronic myeloid leukemia (CML), and BCR-ABL1/Philadelphia (Ph) negative polycythemia vera (PV), essential thrombocythemia (ET), primary myelofibrosis (PMF), and unclassified MPNs [16,43].

### 2.4 4- Chronic myeloid leukemia (CML)

CML is caused by the reciprocal translocation of the BCR gene on chromosome 22q11.2 and the ABL1 gene on chromosome 9q34. This results in the positivity to the Philadelphia (Ph) chromosome and generation of the BCR-ABL1 oncogene. About 90%–98 % of CML patients harbor this mutation [16,28,44,45]. The incidence of CML is consistently stable, with about 0.7–1.0/100000 individuals affected annually, and the diagnosis is more commonly found in individuals within the 60-70-year-old age bracket [16,46,47].

**Fig. 3:**
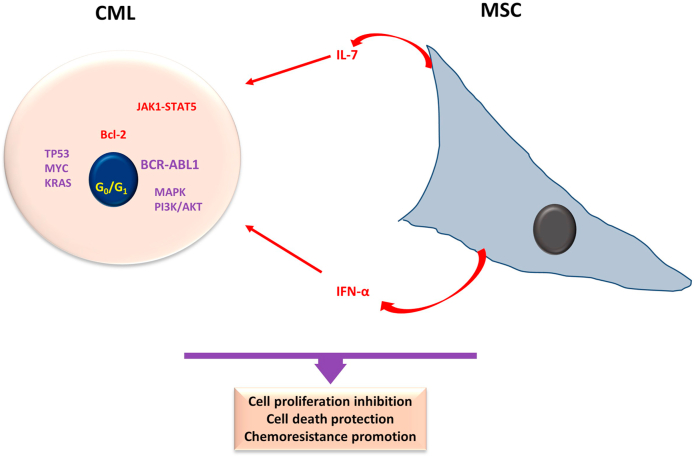
**Main features of Chronic Myeloid Leukemia and Mesenchymal stroma cells.** CML is a type of MPN identified by t(9; 22) *BCR-ABL1* reciprocal chromosomal translocation. The tyrosine kinase coded for by the abnormal *BCR-ABL1* fusion gene provokes hyperactivation of intracellular MAPK and PI3K/AKT signaling and is often accompanied by TP53, MYC, and KRAS gene mutations. MSC may inhibit CML cell proliferation, likely to IFNγ secretion, while protecting cells from cell death and promoting chemoresistance via the IL-7/JAK1/STAT5 axis. Purple indicates the main molecular characteristic of AML, and red indicates the main aspects of MSCs in interaction with the neoplastic cells. CML, chronic myeloid leukemia; MSC. Mesenchymal stroma cells; IFNγ, interferon-gamma; IL-7, interleukin-7; MAPK, mitogen-activated protein kinases. (For interpretation of the references to colour in this figure legend, the reader is referred to the Web version of this article.)

The BCR-ABL1 fusion protein causes the abnormal growth of stem cells due to its overactivation of tyrosine kinase activity [48]. The hyperactivity of tyrosine kinase, independent of cytokines, activates various signal pathways like Ras, MAPK, and PI3K/AKT (Fig. 3), resulting in enhanced growth of leukemic progenitor cells and apoptosis evasion [49,50]. The development of targeted tyrosine kinase inhibitor (TKI) therapy substantially increased the treatment success rates and patient survival, making them an essential tool in the fight against the disease [45,51,52].

Constant cell proliferation favors the occurrence of additional mutations, causing therapy resistance and negatively impacting the disease prognosis [53]. Namely, mutations in TP53, MYC, and KRAS genes, together with immunomodulatory functions of MSCs, support disease progression and refractory response to kinase inhibitors [54,55].

### 2.5 BCR-ABL1/Philadelphia (Ph)-negative myeloproliferative neoplasms

**Fig. 4:**
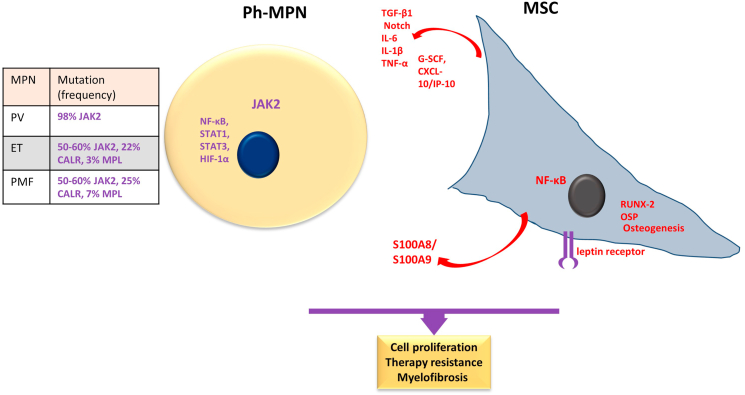
**Main features of Philadelphia (Ph)-negative Myeloproliferative neoplasms and mesenchymal stroma cells.** Philadelphia (Ph)-negative Myeloproliferative neoplasms (Ph-MPN) group of disorders mainly encompasses Polycythemia vera (PV), Essential thrombocythemia (ET), and Primary myelofibrosis (PMF) characterized by mutations in JAK2, CALR, and MPL as shown in the inserted table. In MPN cells, the hyperactivation of JAK2 provokes constitutively activation of NF-ĸB, STAT1, STAT3, and HIF-1α signaling. In turn, MSC promotes MPN cell proliferation and therapy resistance. Moreover, MSC contributes to myelofibrosis due to a profibrotic cytokine milieu, alarmin complex S100A8/S100A9, and leptin receptors also contribute to a bone marrow fibrotic stage Additionally, MSC displays an imbalanced tendency towards osteogenesis differentiation. Purple indicates the main molecular characteristic of AML, and red indicates the main aspects of MSCs in interaction with the neoplastic cells. RUNX-2, runt-related transcription factor-2; OSP, osteopontin; HIF-1α, hypoxia-inducible factor-1; STAT, signal transducer and activator of transcription; NF-ĸB, nuclear factor kappa B; MAPK, mitogen-activated protein kinases. (For interpretation of the references to colour in this figure legend, the reader is referred to the Web version of this article.)

BCR-ABL1 negative MPNs refer to a diverse neoplasm group of the bone marrow multipotent primitive cells that involve the clonal expansion of myeloid lineage in the bone marrow coupled with chronic inflammation [56,57]. This set of neoplasms includes **Polycythemia vera (PV)**, characterized by an elevated red cell count, often accompanied by thrombocytosis; **Essential thrombocythemia (ET)**, diagnosed by megakaryocyte expansion and increased platelet count; and **Primary myelofibrosis (PMF):** Which display extramedullary hematopoiesis, splenic enlargement, and a fibrotic BM [48,56,58,59]. ET or PV can progress to myelofibrosis with bone marrow failure or to secondary acute leukemia [60,61]. The incidence of PV and ET are approximately 0.5–4.0 and 1.1–2.0 cases per 100,000 person-years, respectively, and comparable survival degrees. The incidence of PMF is 0.3–2.0 cases per 100,000 person-years; nevertheless, PMF patients have the shortest survival of the MPNs [62]. The genetic mutations in MPNs have been officially incorporated into the WHO diagnostic criteria for PV, ET, and PMF. PV has a 98 % frequency of JAK2 mutation, while ET has a 50–60 % frequency of JAK2 mutation, 22 % frequency of calreticulin (CALR) mutation, and 3 % frequency of thrombopoietin receptor myeloproliferative leukemia virus (MPL) mutation. PMF has a 50–60 % frequency of JAK2 mutation, 25 % frequency of CALR mutation, and 7 % frequency of MPL mutation (Fig. 4). **Unclassified MPNs:** Additionally, approximately 10–15 % of individuals diagnosed with PMF or ET do not exhibit any of the three primary driver mutations and are also called triple-negative MPNs [48,56,63]. Over 20 other rare mutations have been identified as contributing to disease progression and transformation, including ASXL1 (additional sex combs-like 1), IDH1/2 (isocitrate dehydrogenase 1/2), TP53, and TET2 (TET oncogene family member 2) [64,65].

In MPN, clonal myeloproliferation occurs due to chronic inflammation triggered by factors like infection, hypoxia, or injury or by occurrence of MPN related genetic mutations. It leads to the activation of inflammatory intracellular signal transductions, including the nuclear factor kappa B (NF-ĸB), JAK2-associated STATs effectors, and hypoxia-inducible factor-1 (HIF-1α), generating an increased production of cytokines [66,67]. A crucial aspect of MPN is the dysregulation of the immune system. The accumulation of CD4^+^CD25^+^ FOXP3+ regulatory T-cells (Tregs) and myeloid regulatory cells, along with an imbalanced CD4+/CD8+ T-cell ratio, are proposed as significant immune parameters in the disorder advancement of the refractory response to therapies [68,69].

## 3 Mesenchymal stromal cells

MSCs have shown promising outcomes for cellular treatments and regenerative medicine. These cells possess multilineage differentiation capacities, immunomodulatory properties, and trophic factor expression that can promote tissue repair and regeneration [70,71]. Generating colony-forming unit fibroblasts (CFU–F) in vitro highly defined MSCs and their capacity to assemble a functional bone marrow niche in vivo is essential for generating blood cells [72].

In 2006, the International Society for Cellular Therapy (ISCT) established basic molecular and cellular principles to characterize human MSCs, including the adhesion to plastic surfaces, the expression of CD90, CD73, and CD105 by over 95 % of cells in a given population of MSCs, and the absence of CD45, CD34, CD14, or CD11b, CD79α or CD19, and HLA class II expression. In addition, MSCs, as pluripotent cells, should display osteogenic, adipogenic, or chondrogenic differentiation upon specific inductions in vitro [73,74]. MSCs also have reduced expression of costimulatory molecules such as CD40, CD80, and CD86 [75]. These minimal criteria provided a standardized definition and characterization of MSCs across various research studies and laboratories.

MSCs derived from bone marrow are defined as Lin–CD^45^–CD271^+^CD140^low/–^ fraction. Moreover, these cells are classified based on the CD146 expression. CD146+ MSCs are situated near vasculature and support HSCs by the expression of IGF2, WNT3A, JAG1, CXCL12, KITLG, and ANGPTL, among others [72,[76], [77], [78]]. Meanwhile, nestin-positive, CD146- MSCs enhance HSC's long-term multipotency [[79], [80], [81]].

Although BM has significantly been studied as an MSC supply, the recovery of BM-MSCs requires an invasive surgical procedure. It yields a relatively low number of cells (0.001%–0.01 %), inversely correlated with the donor's age [82]. Interestingly, BM-MSCs can migrate to damaged organs or tissues after being delivered intravenously. Moreover, MSCs from the bone marrow enter the bloodstream and actively migrate to different tissues in response to inflammation or damage. BM-MSCs can also homing and engraftment in solid cancer, such as breast, lung, pancreatic, colon, and prostate carcinomas [82,83].

Because they tended to migrate towards inflammatory sites, MSCs' chemotactic responses are thought to resemble those of immune cells. This is supported by inflammatory cytokines significantly regulating the mobilization, trafficking, and homing of MSCs from the bone marrow to tumor places [81,82].

**Fig. 5:**
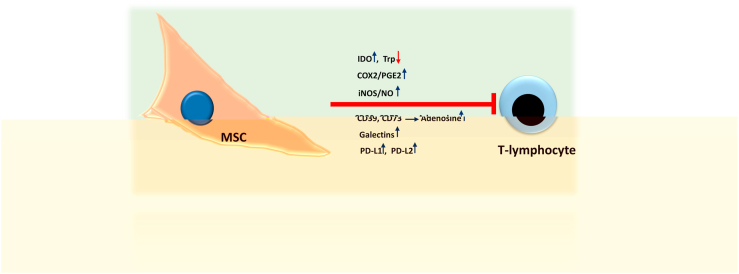
**An overview of the immunosuppressive mechanisms of MSCs:** MSCs have a potent immune-suppressive function on T-cells and may express and release proteins and molecules that inhibit their activation and function. MSC, mesenchymal stroma cells; iNOS, inducible nitric oxide synthase; NO, nitric oxide; COX2, cyclooxygenase-2; PGE2, prostaglandin-2; IDO, indoleamine 2,3-dioxygenase; Trp, tryptophane; CD39, triphosphate diphosphohydrolase-1 and CD73, ecto-5′-nucleotidase; PD-L1, PD-L2, programmed death-ligand 1, -ligand 2.

MSCs exert their immunoregulatory function via cellular-cellular interactions and the production and secretion of several biomolecules, as shown in Fig. 5 [[84], [85], [86]]. MSCs can prevent T lymphocytes from becoming activated inappropriately in cases of inflammation. They can also create an environment that promotes immune tolerance during wound healing. Thereby, MSCs help maintain the immune system's balance.

MSCs regulate almost all the immune system components, thus impacting cancer development and progression [87,88]. For example, MSCs interact with various cell members of the immune system, including natural killer (NK) cells, monocytes/macrophages, dendritic cells, B-lymphocytes, and T-lymphocytes, and significantly influence immune response [84]. Despite their beneficial immunoregulatory properties, they can promote cancer progression and support cancer cells in evading the immune system [[84], [85], [86],^89^,^90^].

## 4 Mesenchymal stroma cells and T-cell immunosuppression

Interestingly, MSCs do not activate alloreactive T-cells in vitro because of reduced HLA class II and costimulatory protein expression; as a result, they are often considered hypoimmunogenic cells [[91], [92], [93]]. When MSCs migrate to sites of inflammation, they regulate innate and adaptive immunity by secreting molecules or via direct interaction with other cells [[93], [94], [95], [96]]. Moreover, there is a consensus that MSCs can suppress T-cell activation by regulating the balance between Th1 and Th2 cells [93,94]. MSCs also impair the activity of cytolytic T-cells [97] (Fig. 5).

Mammal MSCs can be divided based on their use of indoleamine 2,3-dioxygenase (IDO) or inducible nitric oxide synthase (iNOS) for their immunosuppressive functions. Cytokine-licensed MSCs from monkeys, pigs, and humans use IDO, whereas those from mice, rats, rabbits, and hamsters (clade Glires) predominantly use iNOS [98,99].

Human MSCs, via IDO secretion, stimulate INF-γ production that results in the depletion of tryptophan (Trp), which inhibits allogeneic T-cell responses and IFN-γ production by Th1 cells while promoting IL-4 secretion by Th2 cells [[100], [101], [102], [103]]. It seems that Trp depletion is more critical than its metabolite kynurenine accumulation in terms of the inhibitory effects of IDO on T lymphocytes [104,105].

The synthesis of prostaglandin E2 (PGE2) by cyclooxygenase-2 (COX-2) crucially regulates inflammation and cancer's evasion of the immune system [106,107]. In fact, interleukin-6 (IL-6), TNF-α, and IFN-γ enhance PGE2 expression in MSCs [[108], [109], [110]]. Meanwhile, indomethacin inhibition of COX2 significantly improved T-cell proliferation in co-culture approaches using MSCs from BM, adipose tissue, and Wharton's jelly [111,112]. Furthermore, COX2 and PGE2 influence the MSCs' self-renewal and proliferation through an autocrine mechanism involving the prostaglandin EP2 receptor [113].

Besides, MSCs are characterized by the expression of enzymes that convert ATP to adenosine (Ad), such as ectonucleoside triphosphate diphosphohydrolase 1 - CD39 and ecto-5′-nucleotidase - CD73 [114,115]. Ad displays immunosuppressive activity by interacting with its adenosine A2a receptor (ADORA2A), promoting the cytoplasmic cAMP generation and suppressing T-cell activities along with cell anergy [[116], [117], [118]]. MSCs express isoforms 1, 3, and 9 of carbohydrate recognition proteins galectins, which have been shown to induce cell death in active T-cells [[119], [120], [121], [122]].

Furthermore, MSCs can downregulate T-cell activation and responses through direct cell-cell interactions. This is achieved by inducing T-cell cell death via the immune checkpoint proteins programmed death-1 (PD-1) and PD-L1 and PD-L2 [123,124]. Namely, the co-culture of MSCs with autologous splenocytes promotes PD-L1 and PD-L2 generation when stimulated with phytohemagglutinin. However, PD-1, PD-L1, and PD-L2 blocking antibodies enable splenocytes to proliferate under MSC co-cultures [125,126]. Meanwhile, when placenta-derived MSCs are exposed to Interferon-γ and TNF-a, they increase their expression of PD-L2 and can inhibit T cell proliferation stimulated by anti-CD3 antibodies. This effect is accompanied by a shift in T cell differentiation towards a Tregs phenotype. However, when anti-PD-L1 monoclonal antibodies are added, they protect against the inhibition of T-cell proliferation by MSCs obtained from the placenta [127,128].

## 5 Mesenchymal stroma cells in myeloid malignancies

The BMME plays a critical function in cancer progression by either aiding the growth of malignant cells while suppressing healthy blood cell production or undergoing mutations or functional alterations [7,72]. Exposure to hematopoietic cells carrying somatic mutations alters the function of BMME, creating a proinflammatory environment that promotes the development of leukemic disease while suppressing normal hematopoiesis [129]. Namely, it has been observed that there are disparities in the MSC compartments between myeloid cancers and regular bone marrow. The crosstalk between myeloid-transformed cells and MSCs represents a strong relationship influencing leukemia initiation, progression, and response to therapy [130].

### 5.1 Mesenchymal stroma cells and acute myeloid leukemia (AML)

MSCs influence cell viability and favor unresponsiveness to chemotherapy of AML cells (Fig. 1). For instance, MSCs have been shown to exhibit an antiproliferative effect on AML cells. In particular, when U937, HL-60, and multidrug-resistant HL-60/VCR AML cell lines were co-cultured with the human bone marrow stromal cell line (HFCL), they underwent a G_1_ cell cycle arrest. Additionally, the co-culture of HFCL with AML cells prevented cell death induction by the topo I-inhibitor topotecan [131].

In addition, umbilical cord blood-derived MSCs, via IL-6 and IL-8 secretion, have been shown to inhibit AML cell growth by inducing G_0_/G_1_ cell cycle arrest [132]. However, this induction of quiescence and protection from apoptosis can lead to increased chemoresistance in AML cells, associated with increased expression of c-myc and Bcl-2 and activation of Notch signaling [[133], [134], [135]]. Additionally, the mammalian target of rapamycin (mTOR) signaling pathway activation in BM-MSCs has been found to have an anti-apoptotic and growth-promoting effect on AML patient-derived cells [136].

In contrast to normal MSCs, AML-derived BM-MSCs can increase the growth of AML cells. To achieve this, AML-derived BM-MSCs require overexpression of the intracellular Notch domain (Notch ICN), which is essential for increasing AML cell proliferation. Dexamethasone-mediated inhibition of Notch signaling inhibits leukemia and MSCs interplay. Conversely, preventing Notch signaling in AML-BM-MSCs through dexamethasone treatment decreases AML cell proliferation [137].

Studies have shown that direct contact between MSCs and AML cells is essential for developing drug resistance. Garrido et al. [138] demonstrated this in co-culture experiments of patient-derived AML cells with HS-5 human BM stromal cells; patients-derived cells showed resistance to apoptosis when treated with cytosine arabinoside or daunomycin [139]. Moreover, BM-MSCs induce the expression of aldehyde dehydrogenase (ALDH) −1 and −2, generating an ALDH + stem cell-like phenotype in AML cells with enhanced chemotherapy resistance. Besides, stromal cells in the AML-BMME exhibit increased TGF-β expression, which promotes tumor growth and refractory response to chemotherapy through the no-canonical p38 MAPK pathway to increase ALDH2 [140].

Moreover, increased adipogenesis of AML-derived BM-MSCs may contribute to the AML cells' chemotherapy resistance. Specifically, AML-BM-MSCs exhibit lower levels of global m6A and decreased methyltransferase 3 (METTL3) expression than MSCs obtained from healthy donors. This deficiency in METTL3 appears to facilitate AML-BM-MSC adipogenesis through RAC (Rho family)-alpha serine/threonine-protein kinase (AKT)-1 activation, with the potential of development resistance to chemotherapy of AML cells [141].

Patient-derived BM-MSCs show several differences compared to healthy BM-MSCs. Nevertheless, both patients-BM-MSCs and healthy BM-MSCs have similar CD90, CD73, and CD44 expression levels, while AML-BM-MSCs exhibit chemoattractant protein-1 levels reduced expression [142]. Besides, BM-MSCs from AML patients have an increased ability to promote hematogenesis by an altered expression of CD44, CD49e, CD271, and CXCL12 [143,144]. Moreover, around 25 % of AML-BM-MSCs exhibit genetic abnormalities, including chromosomal translocations [145]. Additionally, AML-BM-MSCs have mutations in genes involved in plectin and chromatin remodeling, as well as hypermethylation of Paired Like Homeodomain (PITX)-2 and Homeobox (HOX)B6 genes and hypomethylation of HOXA3 and HOXA5 genes [145,146].

Furthermore, there have been reports of functional changes in AML-BM-MSCs. Some studies suggest that AML-MSCs have diminished clonogenicity and proliferation and are prone to become senescent; however, CFU-F generation is reestablished in AML-BM-MSCs in total remission patients [147,148]. On the other hand, some studies have shown contradictory results, suggesting that AML-BM-MSCs have increased clonogenic potential and immunosuppressive capacity. A study showed that AML-MSCs derived from patients with different risk levels exhibit increased clonogenic potential [149]. Additionally, AML-BM-MSCs have been found to have increased immunosuppressive activities, which may be associated with increased production of the anti-inflammatory cytokine IL-10. Remarkably, inflammatory cytokine expression has also been reduced in AML-MSCs [149]. Furthermore, AML cells secreting IFNγ educate BM-MSCs to induce Treg, which highly contributes to creating an immuno-tolerant BMME [150].

### 5.2 Mesenchymal stroma cells and myelodysplastic syndrome (MDS)

The bone marrow of higher-grade MDS patients has a higher density of BM-MSCs than those with lower-grade MDS or benign hematologic disorders. This increased density is associated with a significantly reduced overall survival rate [151]. BM-MSCs derived from MDS patients proliferate less than healthy MSCs in vitro, suggesting intrinsic growth defects. Both healthy and MDS-MSCs have the ability to increase the proliferation and survival of leukemic cells [152]. Patients-BM-MSCs highly support hematopoietic progenitor clonal growth due to decreased expression of CD44 and CD49e (α5-integrin) and diminished expression of costimulatory molecules CD40, CD80, and CD86 [143,[153], [154], [155]]. This suggests that dysfunctional MDS-BM-MSCs lead to abnormal blood cell production [11].

In vitro, MDS-BM-MSCs display modifications in the expression of various cytokines, including reduced production of stem cell factor (SCF), granulocyte cell stimulating factor (G-CSF), and granulocyte-macrophage colony-stimulating factor (GM-CSF), and augmented IL-6 production [156]. They also show altered expressions of adhesion molecules, such as CD44 and CD49e, and molecules involved in the interaction with HSCs, such as osteopontin (OPN), Jagged 1, Kit-L, and Ang 1 [157,158]. The C-X-C motif chemokine ligand 12 (CXCL12) chemokine, associated with the MDS cell viability and clinical disease course, also increases in MDS-MSCs [159]. Furthermore, MDS-BM-MSCs have reduced CFU-F clonogenic potential and proliferation at diagnosis, and exhibit accelerated senescence compared to healthy BM-MSCs [157,[160], [161], [162]]. In vitro, MDS-BM-MSCs promote the myeloid cell lineage of HSCs to the detriment of erythroid differentiation [158]. Furthermore, MDS-BM-MSCs reduce the expression of delta-like 1 (DLK1) protein, an early adipocyte cell fate inhibitor, resulting in an increased number of mature adipocytes in vitro [163,164].

MDS cells secrete molecules that can alter BM-MSC, as shown by the ability of soluble factors from human MDS cells to influence BM-MSCs to develop molecular features similar to those of MDS-MSCs. This includes changes in DNA methylation patterns, increased cytokines and inflammatory factors gene expression, and decreased expression of genes related to the cell cycle promotion, which reduces support for healthy HSCs [147,165]. There are conflicting findings regarding the immunoregulatory function of MDS-BM-MSCs. Some studies suggest that MDS-BM-MSCs have impaired immunoregulatory function, while others suggest no significant difference compared to healthy BM-MSCs [160,166,167]. Additionally, MDS-BM-MSCs are immunosuppressive due to the increased production of prostaglandins via COX2, which can reduce T-cell immunity against leukemic cells [168] (Fig. 2). Furthermore, MDS-BM-MSCs may transfer healthy mitochondria into AML cells, significantly increasing cell energy production and enhancing cell viability during clinical treatment [169,170].

### 5.3 Mesenchymal stroma cells and chronic myeloid leukemia (CML)

MSCs are crucial in regulating CML cell proliferation, apoptosis, and resistance to chemotherapy (Fig. 3). Namely, BM-MSCs induce CML cell resistance to imatinib treatment-induced apoptosis by secreting interleukin-7 (IL-7) and activating the JAK1-STAT5 signaling. It is important to mention that in the CML patients' blast crisis phase, the IL-7 expression is likely to rise in the bone marrow [171]. BM-MSC can also improve Imatinib therapy's effectiveness by increasing CML cell cycle arrest in the G_0_/G_1_ phase. Imatinib may promote the migration of CML cells to BM by enhancing CXCR4 expression and hindering cell proliferation [172]. Consequently, BM-MSC may safeguard and boost the CML cell viability in a quiescent state, potentially leading to disease recurrence [173,174].

The contact with BM-MSCs inhibits cell death and induces a state of quiescence in the Ph + human BV173 and K562 cell lines, in parallel with a decrease in cyclin-D2. On the other hand, MSCs encourage xenograft development of CML cells in nonobese diabetic/severe combined immunodeficiency mice by preventing apoptosis. MSCs could create a microenvironment that supports the CML stem cells' self-renewal and maintains the malignant processes [175]. Moreover, BM-MSCs impede the proliferation of co-cultivated CML mononuclear cells in vitro by producing increased levels of interferon (IFN)-α. It is worth noting that IFN-α was the therapy of choice for CML before introducing tyrosine kinase inhibitors [176,177]. Additionally, there have been findings that BM-MSCs extracted from CML patients in the blastic phase can provide protection against apoptosis caused by Adriamycin in primary CML Bp cells. This is achieved through decreased caspase-3 and Bax expression, increased Bcl-2 levels, and Wnt pathway activation [178].

### 5.4 Mesenchymal stroma cells in Ph-myeloproliferative neoplasms (Ph-MPN)

MSCs can contribute to the proliferation of neoplastic myeloid cells and promote the development of BM fibrosis. Specifically, in a mouse model of primary myelofibrosis with elevated levels of thrombopoietin (Tpo), MSCs that express the leptin receptor are responsible for forming myofibroblasts within the BM, which is a critical factor in the disease progression [179]. Currently, there are no visible differences in the appearance, growth rate, and ability to transform into different cell types between MSCs derived from individuals with MPN (JAK2^V617F^) and those without the condition. However, the MNP-derived BM-MSCs are more effective at promoting the survival and growth of CFU-GM colonies in MPN-HSC. PV and ET patients-derived MPN-MSCs consistently display altered expression patterns of genes linked to healthy blood cells, including overexpression of SPP1 and NF-κB and downregulation of angiopoietin-1 and thrombopoietin. As a result, BM-MSCs from patients may promote MPN cell growth and preservation of neoplastic features [180].

Current literature reports no visible differences in the appearance and growth rate between BM-MSCs derived from individuals with MPN (JAK2^V617F^) and those without the mutation. For instance, Schneider et al. [181] have revealed that ET and PV-derived BM-MSCs have characteristics similar to healthy MSCs regarding surface markers, morphology, CFU-F clonal differentiation, and differentiation potential. Interestingly, ET-derived BM-MSCs exhibited reduced secretion levels of G-CSF and IL-7, which could be accounted for decreased hematopoiesis. Additionally, supernatants from PV cells were found to reduce normal myeloid CFU when cultivated [181]. PMF-derived BM-MSCs exhibit similar proliferation rates and support the formation of blood cellular components comparable to normal BM-MSCs. However, PMF, PV, and ET-BM-MSCs appear to have undergone reprogramming, making them more inclined towards osteogenic differentiation than normal BM-MSCs [182].

In addition, patients-derived BM-MSCs can cause an aberrant production of 'myelofibrotic' cells due to an unstable inflammatory environment caused by increased TGF-β1, Notch, IL-6, IL-1β, and TNF-α expression [183]. Furthermore, an overabundance of inflammatory 'myelofibrotic cells' promotes a fibrotic BM in the late MPN phases. Additionally, an aberrant number of osteoblasts promotes the continuous proliferation of clonal-MPN cells [184,185].

MSCs contribute to the myelofibrotic phenotype in PMF. Specifically, in a mouse model of primary myelofibrosis with elevated levels of thrombopoietin (Tpo), MSCs that express the leptin receptor are responsible for forming myofibroblasts within the bone marrow, which is a critical factor in the disease progression [179]. In patients with myeloproliferative neoplasms and murine models, the expression of the alarmin complex S100A8/S100A9, a crucial proinflammatory player in MPNs, in MSC mediates the disease progression toward the fibrotic phenotype. The increased alarmin complex expression is linked to a functional reprogramming of MPN-MSC to a fibrotic stage with harm to their progenitor features and normal functionality in the BM niche. The inhibition of S100A8/S100A9 by Tasquinimod, a small oral inhibitor, ameliorates the MPN phenotype and fibrosis, likely by affecting the mutated hematopoietic clone and MSC fibrotic transformation [186]. Moreover, the inflammatory JAK2/STAT3 signaling pathway significantly contributed to TGF-β-induced fibrosis of BM-MSC. In this sense, combined inhibition of JAK1/2 and SMAD3 synergistically mitigates BM-MSCs fibrosis [187].

Remarkably, human MPN and mice harboring HSCs with JAK2^V617F^ display decreased BM innervation, including sympathetic nerve fibers and Schwann cells, and reduced nestin + MSCs. Thus, eliminating nestin + MSCs in the bone marrow with sympathetic innervation seems necessary for developing MPN.

The mutant myeloid cell expansion can be prevented by restoring sympathetic regulation of nestin + MSCs through β3-adrenergic agonists [188]. IL-6, G-SCF, and CXCL-10/IP-10 production by PV-derived BM-MSCs contribute to the refractory response to JAK2 inhibition treatment. It reduces JAK2^V617F^ myeloid cell death when cultured in the presence of MSCs in vitro [189] (Fig. 4).

## 6 Therapeutic perspectives

**Fig. 6:**
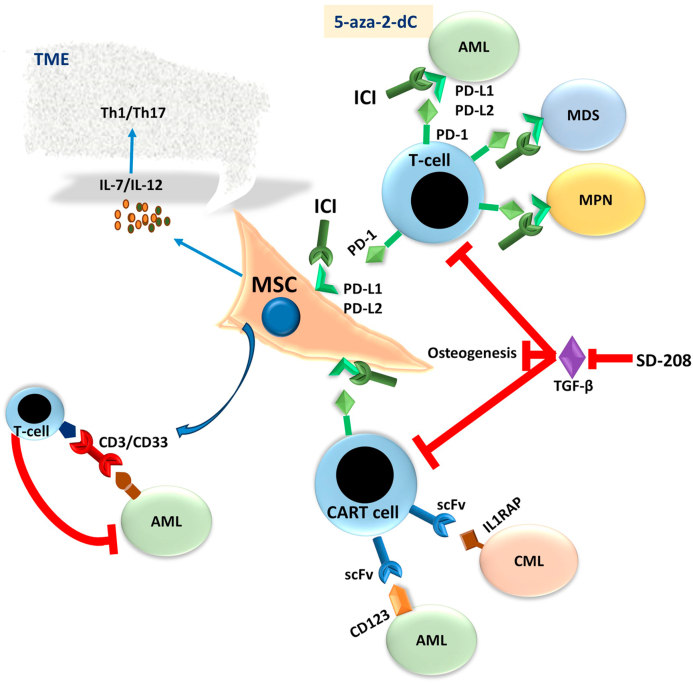
**Mesenchymal stroma cells and myeloid malignancy therapies:** MSCs are being targeted for developing new therapeutic strategies to enhance current treatments due to their involvement in myeloid neoplasm pathogenesis. The figure summarizes the main aspects of MSC implications in myeloid malignancy therapy discussed in the main text, such as influencing tumor microenvironment (TME) by IL-7 and IL-12 secretion, TGF-β inhibition, chimeric antigen receptor T (CART) and immune checkpoint inhibitors (ICI). MSC, mesenchymal stroma cells; AML, acute myeloid leukemia; MDS, myelodysplastic syndrome; CML, chronic myeloid leukemia; MPN, Philadelphia chromosome-negative myeloproliferative neoplasms.

Due to their involvement in myeloid neoplasm pathogenesis, MSCs have become a target for developing new therapeutic strategies that could enhance current treatments [[190], [191], [192], [193]] (Fig. 6). For instance, MSCs genetically modified with anti-CD33^−^CD3 bifunctional antibodies genes may revoke the onset of AML in nonobese diabetic/severe combined immunodeficiency IL2Rγ−/− (NSG) mice and redirect allogeneic T-cells against AML patient-derived blasts [194,195].

Notably, TGF-β1 can cause healthy MSCs to exhibit dysfunctional phenotypes resembling those seen in stromal cells derived from patients with myeloid neoplasms. However, the TGF-β inhibitor SD-208 restores MSC healthy functions and osteogenesis of AML/MDS-MSCs [196]. Research indicates that TGF-β1 levels are increased in myeloid neoplasms [197,198]. It is possible that inhibiting TGF-β could improve the function of MSCs in the bone marrow niche. Furthermore, blocking TGF-β may enhance T-cell function, as it has a robust immunosuppressive effect [199].

Enhancing the anticancer T-cell activity in myeloid malignancies through reprogramming the immune dysregulation is a promising immunotherapeutic strategy. Studies have demonstrated the effectiveness of immune checkpoint inhibitors (ICIs) and chimeric antigen receptor T (CART) therapies. Immune checkpoints are crucial in regulating T-cell antigen responses. However, ICIs amplify the killer cells' cytotoxic capabilities against myeloid neoplastic leukemic cells, which showcases the potential of using ICIs to boost the immune system's capacity to combat myeloid neoplasms [[200], [201], [202]].

Activated T cells can induce the immune checkpoint PD-1, which, when bound with PD-L1, can produce functionally inactivated T-cells [203]. Remarkable outcomes have been seen in treating solid tumors and hematological disorders through anti-PD-1 and anti-PD-L1 blocking antibodies, such as AML and MDS [[204], [205], [206]]. When tested, especially in combination with DNA methyltransferase inhibitor agents like 5-aza-2′deoxycitidine, these immune checkpoint blockers have improved outcomes on chemotherapy-resistant AML treatments [206]. Furthermore, it has been found that the lead of JAK2V617F MPN cells has increased PD-L1 expression and escape of immunosurveillance capabilities [207]. As a result, several clinical studies investigate the ICIs' lack of side effects and success in MPN treatments (Reviewed in 59 and references therein). Additionally, MSCs are potential targets for ICIs due to their expression of PD-L1/PD-L2, which suppresses T-cell activation and responses and can trigger T-cell apoptosis [123,124,208,209]. Therefore, ICIs immunotherapy could potentially hinder the immunosuppression of MSCs and improve the killer T-cell elimination of myeloid neoplasic cells.

A promising treatment for myeloid malignancies is the chimeric antigen receptor T (CART) cells adoptive cellular immunotherapy [202,210,211]. CARs are genetically modified receptors composed of an extracellular domain, a transmembrane domain, and an intracellular T cell activation and co-stimulation signaling domain. The extracellular domain comprises a single-chain variable fragment (scFv) derived from an antitumor antigen-antibody. The intracellular domain primarily contains CD3ζ, CD28, and 4-1BB [[212], [213], [214]]. To carry out CART cell immunotherapy, autologous T-cells are genetically modified to express a specific CAR, expanded in vitro, and then reinfused into the same patients to eliminate neoplastic cells.

AML therapeutic strategies involving CART targeting the expression of IL-3Rα (CD123) have shown promising results. The antitumor activity of CD123-CART cells has been observed in both in vitro and in vivo settings against CD123+ AML cell lines and patients' AML cells. Moreover, CD123-CART therapy has no significant toxicity on healthy HSCs, demonstrating the safety of the treatment. The CD123 CART first-in-human clinical trial has shown promising results regarding AML remissions while maintaining acceptable feasibility and safety [[215], [216], [217], [218]]. Also, it has been observed that CART cells could effectively target IL1 receptor-associated protein (IL1RAP) within inactive CML stem cells [219,220]. IL1RAP-CART cells have been found to react positively against IL1RAP + cell lines or patient-derived CML cells. They have demonstrated the ability to produce proinflammatory cytokines and eliminate neoplastic cells in mouse xenograft models. Notably, IL1RAP-CART-cells can specifically destroy transformed cells, keeping healthy CD34^+^ stem cells unaltered [221]. However, using CART to treat myeloid malignancies poses a challenge as it needs specific antigen expression on the outer face of the plasma membrane of malignant cells [204,211].

Currently, CART cell strategies focus on targeting leukemic blasts. Nevertheless, MSCs hinder T-cell activities, making it promising to combine the targeting of the immunomodulatory MSC function with CART-based adoptive cellular approaches to enhance current therapies for hematological malignancies [222]. It is important to point out that modifying MSCs genetically to increase the expression of IL-7 and IL-12 can alter the chronic inflammatory milieu into a more anti-tumor-responsive Th1/Th17 TME. This change leads to an acute activation of CART cells and enhances the antitumor response [223].

## 7 Concluding remarks

Myeloid malignancies involve several cellular and genetic alterations in hematopoietic cells and their surrounding TME, including MSC, creating a driving environment for tumor progression. The current review highlighted the role of MSCs as reactive cells that can facilitate expansion, safeguard myeloid malignant cell viability, and decrease the effectiveness of chemotherapy and immunotherapies approaches. MSCs have the potential to serve as predictor cellular markers for the response to the therapies and the likelihood of disease recurrence. These cells are also valuable for therapeutic strategies in combination with available clinical treatments and promising novel CART and ICIs immunotherapies. By comprehending MSCs' function in myeloid malignancies, it is possible to create innovative ways to generate improved and personalized medicine to cure and enhance patients' quality of life.

## Data availability statement

No datasets were generated or analyzed during the current study.

## CRediT authorship contribution statement

**Milica Vukotić:** Writing – original draft. **Suncica Kapor:** Writing – original draft. **Felipe Simon:** Writing – review & editing, Conceptualization. **Vladan Cokic:** Writing – review & editing. **Juan F. Santibanez:** Writing – review & editing, Conceptualization.

## Declaration of competing interest

The authors declare that they have no known competing financial interests or personal relationships that could have appeared to influence the work reported in this paper.
